# Draft Genome Sequence of Hexachlorohexane (HCH)-Degrading *Sphingobium lucknowense* Strain F2^T^, Isolated from an HCH Dumpsite

**DOI:** 10.1128/genomeA.00788-14

**Published:** 2014-08-07

**Authors:** Vivek Negi, Pushp Lata, Naseer Sangwan, Sanjay Kumar Gupta, Shreyasi Das, D. L. N. Rao, Rup Lal

**Affiliations:** a Department of Zoology, University of Delhi, Delhi, India; b Indian Institute of Soil Science Nabi Bagh, Bhopal, Madhya Pradesh, India

## Abstract

*Sphingobium lucknowense* F2^T^, isolated from the hexachlorocylcohexane (HCH) dumpsite located in Ummari village, Lucknow, India, rapidly degrades HCH isomers. Here we report the draft genome of strain F2 (4.4 Mbp), consisting of 4,910 protein coding genes with an average G+C content of 64.3%.

## GENOME ANNOUNCEMENT

To continue our previous efforts to characterize the microbial diversity at a hexachlorocyclohexane (HCH) dumpsite by using culture-dependent (1–6) and culture-independent (7, 8) approaches, we have isolated from an HCH dumpsite yet another bacterial strain, characterized as *Sphingobium lucknowense* F2^T^ (1). This strain was found to rapidly degrade all HCH isomers (α-, γ-, β-, and δ-HCH), as reported earlier for *Sphingobium* spp. (1, 9–12). This motivated our efforts to sequence the genome of F2^T^ and compare its draft genome with the already-sequenced draft genomes of sphingomonads isolated from a similar HCH dumpsite (13–19).

The draft genome sequence of strain F2 was generated by using Illumina HiSeq 2000 (~5,744,444 paired-end reads) and 454 GS FLX titanium platforms (~77,766 single reads). The sequence data were assembled using Velvet_1.2.03 (20) at a k-mer length of 59 (expected coverage, 50; coverage cutoff, 10; and minimum contig length cutoff, 500 bp), ABySS 1.3.4 (21) at a k-mer length of 49, and SPAdes 3.0 (22). The sets of contigs generated from these assemblers were subjected to hybrid assembly integrated by Contig Integrator for Sequence Assembly (CISA) (23). We obtained 103 contigs having a total size of 4.4 Mbp, with average GC content of 64.3%. RNAmmer 1.2 (24) and ARAGORN (25) were used to predict rRNA genes, i.e., 2 5S rRNAs, 1e 16S rRNA, 1 23S rRNA, 49 tRNA, and 1 transfer-messenger RNA (tmRNA) gene. The Rapid Annotations using Subsystems Technology (RAST) pipeline (26), the KEGG Automatic Annotation Server (KAAS) (27), and the NCBI Prokaryotic Genomes Automatic Annotation Pipeline (http://www.ncbi.nlm.nih.gov/genomes/static/Pipeline.html) were used for annotation. RAST annotation revealed 4,910 coding sequences (CDSs), of which 41.85% have hypothetical proteins. Four insertion elements (IS) (IS*1247*, IS*6100*, IS*Shsp3*, and IS*Ssp5*) were identified on the basis of their sequence length and different layouts. The draft genome was binned using a Hidden Markov model (HMM) profile of 107 essential single-copy genes conserved in 95% of all bacteria, and it was found to contain 97 such genes, suggesting more than 90.7% completion (28).

*Sphingobium lucknowense* F2^T^ contains one copy each of *linA*, *linB*, *linC*, *linD*, *linE*, *linF*, *linI*, *linJ*, *linK*, *linL*, *linM*, *linN*, and *linR* and four copies of *linG* and *linH*. These *lin* genes have already been implicated in the degradation of HCH isomers (10, 29, 30). Three putative gene clusters involved in degradation of chlorophenol, homogentisate, and toluene/phenol in other sphingomonads (14, 19) were also identified in the draft genome of *Sphingobium lucknowense* F2^T^.

Strikingly, strain F2^T^ was found to contain higher abundances of membrane transport proteins (*n* = 228), stress response proteins (*n* = 117), putative aromatic hydrocarbon pathways (*n* = 30), and transposable elements and proteins (*n* = 12) of phages and prophages. The draft genome sequence of F2^T^, along with the genome sequences of seven HCH-degrading *Sphingobium spp.* (13–17, 30, 31) and metagenome data from the HCH dumpsite (7, 8) will now provide deeper insights into the evolution and acquisition of *lin* genes within this group of sphingomonads.

### Nucleotide sequence accession numbers.

The genome sequence of *Sphingobium lucknowense* F2^T^ has been assigned the GenBank accession number JANF00000000. The version described in this paper is version JANF02000000.
